# AMF Inoculation Can Enhance Yield of Transgenic *Bt* Maize and Its Control Efficiency Against *Mythimna separata* Especially Under Elevated CO_2_

**DOI:** 10.3389/fpls.2021.655060

**Published:** 2021-06-08

**Authors:** Long Wang, Xiaohui Wang, Fanqi Gao, Changning Lv, Likun Li, Tong Han, Fajun Chen

**Affiliations:** ^1^Department of Entomology, College of Plant Protection, Nanjing Agricultural University, Nanjing, China; ^2^Department of Landscape Architecture, College of Biological and Agricultural Engineering, Weifang University, Weifang, China; ^3^Jinshanbao Experimental Class, College of Agriculture, Nanjing Agricultural University, Nanjing, China; ^4^Department of Phytology, College of Life Sciences, Nanjing Agricultural University, Nanjing, China

**Keywords:** elevated CO_2_, transgenic *Bt* maize, arbuscular mycorrhizal fungi, *Mythimna separata*, control efficiency, yield

## Abstract

The promotion and application of transgenic *Bt* crops provides an approach for the prevention and control of target lepidopteran pests and effectively relieves the environmental pressure caused by the massive usage of chemical pesticides in fields. However, studies have shown that *Bt* crops will face a new risk due to a decrease in exogenous toxin content under elevated carbon dioxide (CO_2_) concentration, thus negatively affecting the ecological sustainability of *Bt* crops. Arbuscular mycorrhizal fungi (AMF) are important beneficial microorganisms that can effectively improve the nutrient status of host plants and are expected to relieve the ecological risk of *Bt* crops under increasing CO_2_ due to global climate change. In this study, the *Bt* maize and its parental line of non-transgenic *Bt* maize were selected and inoculated with a species of AMF (*Funneliformis caledonium*, synonyms: *Glomus caledonium*), in order to study the secondary defensive chemicals and yield of maize, and to explore the effects of *F. caledonium* inoculation on the growth, development, and reproduction of the pest *Mythimna separata* fed on *Bt* maize and non-*Bt* maize under ambient carbon dioxide concentration (*a*CO_2_) and elevated carbon dioxide concentration (*e*CO_2_). The results showed that *e*CO_2_ increased the AM fungal colonization, maize yield, and foliar contents of jasmonic acid (JA) and salicylic acid (SA), but decreased foliar Bt toxin content and *Bt* gene expression in *Bt* maize leaves. *F. caledonium* inoculation increased maize yield, foliar JA, SA contents, *Bt* toxin contents, and *Bt* gene expression in *Bt* maize leaves, and positively improved the growth, development, reproduction, and food utilization of the *M. separata* fed on non-*Bt* maize. However, *F. caledonium* inoculation was unfavorable for the fitness of *M. separata* fed on *Bt* maize, and the effect was intensified when combined with *e*CO_2_. It is indicated that *F. caledonium* inoculation had adverse effects on the production of non-*Bt* maize due to the high potential risk of population occurrence of *M. separata*, while it was just the opposite for *Bt* maize. Therefore, this study confirms that the AMF can increase the yield and promote the expression levels of its endogenous (JA, SA) and exogenous (Bt toxin) secondary defense substances of *Bt* maize under *e*CO_2_, and finally can enhance the insect resistance capacity of *Bt* crops, which will help ensure the sustainable utilization and safety of *Bt* crops under climate change.

## Introduction

In recent years, many transgenic *Bt* crops, such as *Bt* maize and *Bt* cotton, have been grown around the world and have shown high resistance to specific target pests, mainly Lepidoptera insects (Wu et al., 2008; Liu et al., 2016). As a result, *Bt* crops have been used to control a wider range of pests, such as *Helicoverpa armigera* (Hübner), *Heliothis virescens*, and *Mythimna separata* (Riddick et al., 1998; Chen F. J. et al., 2011; Chang et al., 2013). Meanwhile, human activities, specifically fossil fuel burning and land-use change, are rapidly increasing the level of carbon dioxide (CO_2_) in the atmosphere (Yu and Chen, 2019; Yao et al., 2020). Specifically, it has been reported that the atmospheric CO_2_ concentration increased from 288 to 405 ppm from 1800 to 2018 (www.esrl.noaa.gov/gmd/ccgg/trends/). With the acceleration of industrialization, it is estimated that the concentration of CO_2_ in the atmosphere will increase from 800 to 1,000 ppm by the end of the twenty-first century (Pachauri and Reisinger, 2014).

Plant productivity is fundamentally tied to atmospheric CO_2_ by photosynthesis, and the increase in atmospheric CO_2_ concentration can improve the photosynthetic capacity of plants and undoubtedly affect the plant physiology with profound impacts on all aspects, including the increase in photosynthetic rate, biomass, and seed production (Dietterich et al., 2015; Johnson and Hartley, 2018; Zhu et al., 2018). Most studies reported that elevated CO_2_ (*e*CO_2_) increased the C/N ratio in plant tissues; thus, the content of carbohydrates in plant tissues increased and the content of N-containing compounds decreased (Chen et al., 2005a; Xu et al., 2015; Dai et al., 2018). All these changes in turn affect the production of plant secondary metabolites (Stiling and Cornelissen, 2007). Elevated CO_2_ may enhance or weaken plant defense against herbivorous insects, at least partly due to the changes in C- and N-based defensive metabolites, as well as plant nutrients, especially protein content (Kretzschmar et al., 2009). Some studies have shown that jasmonic acid (JA), ethylene (ET), and salicylic acid (SA) are secondary defense substances of plants against aphids under *e*CO_2_ (Sun et al., 2013; Guo et al., 2014), and reported that *e*CO_2_ caused a significant reduction of N-based compounds (i.e., *Bt* toxin proteins) in *Bt* crops (Chen et al., 2005b; Wu et al., 2011a; Liu et al., 2019). Hence, it is speculated that under the condition of climate change, transgenic *Bt* crops will face a new risk that the effective control of the target pests will be reduced.

Arbuscular mycorrhizal fungi (AMF) can form associations with the roots of about 80% of terrestrial plant species (Smith and Read, 2008) and exchange soil-derived nutrients (Marschner and Dell, 1994) for plant-derived hexoses and lipids (Helber et al., 2011; Keymer and Lankau, 2017). AMF improve the supply of inorganic nutrients, especially phosphate (Rillig and Mummey, 2006). However, since AMF can also enhance the nitrogen uptake and utilization of plants (Hawkins et al., 2000) and improve their resistance to external biotic and abiotic stresses (Jung et al., 2012; Frew and Price, 2019), we hypothesize that they can be used to alleviate the problems of *Bt* toxin protein decline under *e*CO_2_, and reduce the risk of *Bt* crops under future climate change. In order to test this hypothesis, we inoculated *Bt* maize with AMF (*Funneliformis caledonium*) under elevated CO_2_ concentration to explore the interaction between *e*CO_2_ and AM fungal inoculation on plant growth and secondary defense metabolites of *Bt* maize, and the effects on the growth and development and food utilization of the main maize pest armyworm from 2017 to 2018. We further hypothesize that *F*. *caledonium* inoculation under *e*CO_2_ could (a) increase the biomass and yield of maize; (b) promote the expression of endogenous (JA, SA) and exogenous (Bt toxin) secondary defense substances in *Bt* maize leaves; and (c) decrease indices of growth, development, and reproduction of *M. separata*.

## Materials and Methods

### CO_2_ Setting

A 2-year experiment (2017–2018) was conducted in six open-top chambers (OTCs) in Ningjin County, Shandong Province of China (37.64° N, 116.8° E). OTCs are 2.5 m in height × 3.2 m in diameter. Two concentration levels of CO_2_ are applied successively, namely, the ambient level (*a*CO_2_, 375 μl/l) and the elevated level (*e*CO_2_, 750 μl/l). Each CO_2_ treatment uses three OTCs. During the experiment, OTCs were continuously filled with CO_2_. The average CO_2_ concentration is shown in Supplementary Table 1.

### Arbuscular Mycorrhizal Fungi and *Bt* Maize

*Funneliformis caledonium* (strain number 90036, referred to as FC, synonyms: *Glomus caledonium*) was provided by the State Key Laboratory of Soil & Sustainable Agriculture, Institute of Soil Science, the Chinese Academy of Sciences. The inoculum consisted of spores, mycelium, maize root fragments, and soil (storage: under normal temperature, keep in dry, cool place). The *Bt* maize cultivar (line IE09S034 with *Cry1Ie*, namely, *Bt*) and the parental line of non-transgenic *Bt* maize (cv. Xianyu 335, namely, Xy) were provided by The Institute of Crop Sciences of the Chinese Academy of Agricultural Science. *Bt* maize and non-*Bt* maize were planted in plastic barrels (the height of 45 cm and the diameter of 30 cm) filled with 20 kg soils and 10 g compound fertilizer (N: P: K = 18: 15: 12), respectively. Soil pH was 7.2, organic carbon 11.7 g/kg, total nitrogen 2.27 g/kg, and total phosphorus 0.56 g/kg. On June 10 of each sampling year, 100 g inoculum of *F. caledonium* (namely, FC in figure) and 100 g sterilized strain (namely, CK in figure) were evenly spread at 4 cm under maize seed as control; three maize seeds were sown in each barrel with a sowing depth of 2 cm, and two maize were reserved after emergence. Maize were irrigated every two to three days to ensure the water demand for maize growth.

There were eight treatments [two CO_2_ concentration (aCO_2_ and eCO_2_), two maize treatments (*Bt* and Xy), and two AMF inoculations (*F. caledonium* and CK)]. Each treatment included three OTCs; each OTC included four planting patterns (*Bt* + AMF, *Bt* + CK, Xy + AMF, and Xy + CK); each planting pattern included five repeats; that is, each treatment contained a total of 15 repeats.

### Arbuscular Mycorrhizal Fungi Colonization and AMF Phospholipid Fatty Acid (PLFA) Content

In two sampling years, AMF colonization was determined on heading stage (BBCH-59), and it was determined by the method of trypan blue staining and grid counting (Phillips and Hayman, 1970). The fresh plant roots were washed with distilled water and then blotted dry with absorbent paper. One hundred one centimeter roots were randomly cut and placed in a 10% KOH solution at 30°C for 30 min, and then, the KOH was discarded and rinsed with distilled water. After acidification in 2% HCl for 60 min, the HCl was discarded, rinsed with distilled water, and stained in 5% trypan blue dye solution (w/v, lactic acid: glycerol: water = 1: 1: 1). Then, the dye solution was discarded, and the roots were rinsed with distilled water and transferred to a square with a grid at the bottom. We observed the number of infected and uninfected root segments under the microscope. Colonization (%) = number of infected root segments/total root segments (Mcgonigle et al., 1990). The improved Bligh-Dyer method was used to extract microbial PLFA from soil (Bossio and Scow, 1998; Ruess and Chamberlain, 2010). About 8.0 g of the freeze-dried soil sample was weighed into a Teflon tube, and the lipids from soil were extracted by multiple oscillation centrifugation with a Bligh-Dyer mixed extract prepared in a ratio of 1: 2: 0.8 with chloroform: methanol: citrate buffer. The aqueous phase and the organic phase were separated by keeping away from light overnight, and the lower organic-phase supernatant containing the phospholipid was taken up and then dried with nitrogen in a water bath. The sample was dissolved and dried with a small amount of chloroform and acetone several times and passed through a SPE silica gel column to remove neutral lipids and glycolipids from the sample, followed by separation and purification with methanol to collect PLFA, and dried with nitrogen again. The separated PLFA is methylated by a liquid (methanol: toluene 1:1 mixture) and a liquid of KOH–methanol solution, and then the reaction was terminated with acetic acid. Finally, it was extracted with n-hexane and dried with nitrogen to obtain PLFA methyl ester. With C19: 0 as the internal standard, the content of methyl ester of characteristic fatty acids was analyzed by MIDI identification system, and the soil PLFA content was expressed in nmol/g through peak area and internal standard curve.

### Foliar Bt Toxin Content and *Bt* Gene Expression in the Leaves of *Bt* Maize

During the heading stage (BBCH-59) of maize, the foliar content of Bt toxin protein was measured by using plant *Bt*-*Cry1Ie* protein ELISA Kit (mlbio, China). Moreover, the real-time quantitative reverse transcription PCR was performed on a 7500 real-time PCR system (Applied Biosystems Inc.) for *Bt* gene expression analysis. Total RNA was extracted from the leaf tissues by using TRIzol® reagent (Invitrogen). The concentration and quality of samples were determined by NanoDrop™ spectrophotometer (Thermo Scientific) and 1.5% agarose gel electrophoresis. The cDNA synthesis was carried out with 100 ng of total RNA by using PrimeScript™ RT reagent Kit with gDNA Eraser (Takara, Japan). Reverse transcriptase reactions were performed in a reaction volume of 20 μl. Quantitative real-time PCR was performed with a 7500 real-time PCR detection system (Applied Biosystems) using 1 × SYBR® *Premix Ex Taq*™ (TaKaRa, Japan), 2 μl cDNA products (diluted from 20 to 200 μl with RNase-free water), and 0.2 μM primers in a final volume of 20 μl. Reaction conditions are 95°C, 30 s pre-denaturation; 95°C, 5 s, 60°C, 34 s, 40 cycles. The cDNA was amplified by PCR using the primers shown in Supplementary Table 2. Quantification of the transcript levels of target genes was conducted by following the 2^−Δ*ΔCt*^ normalization method. The relative expression level was represented as the fold change by comparing three treatments (*a*CO_2_ + AMF, *a*CO_2_ +CK, and *e*CO_2_ +AMF) and the treatment of *e*CO_2_ + CK, respectively. Three technical replicates were performed on each sample of cDNA.

### Jasmonic Acid and Salicylic Acid Contents in Maize Leaves

During the heading stage (BBCH-59) of maize, the foliar contents of JA and SA were measured in our laboratory by using plant JA ELISA Kit (YaJi Biological, China) and plant SA ELISA Kit (YaJi Biological, China).

### Insect Development and Food Utilization

The colony of armyworm, *M. separata*, was collected in maize fields in Kangbao County, Hebei (China), and continuously reared on artificial diet for more than 15 generations in growth chamber (GDN-400D-4; Ningbo Southeast Instrument CO., LTD, Ningbo, China) (Song et al., 2020). The third-instar larvae with uniform size were randomly selected and were individually fed on fresh maize leaves, which were selected from each treatment at the heading stage (BBCH-59). The feeding trials were conducted in a plastic dish (6 cm in diameter) in 2017 and 2018. Each treatment consisted of five replicates (a total of 20 larvae per replicate).

The initial weights of third-instar larvae were individually measured with an electronic balance (AL104; METTLER-TOLEDO, Greifensee). Larvae feces, pupal weight, and the remaining parts of leaves were also carefully weighed. At the same time, dry weight of larvae and maize leaves was calculated during the experiment. Moreover, the food utilization indices, including relative consumption rate (RCR), relative growth rate (RGR), efficiency of conversion of digested food (ECD), and efficiency of conversion of ingested food (ECI), were measured (Chen et al., 2005b).

$$\begin{array}{l} RCR=\text{ I/(B*T); RGR}=\text{G/(B*T);}\\ \text{ECD (​​}\%\text{​​)}=\text{G/(I-F)*100​​}\%\text{;​​ECI (​​}\%\text{​​) =G/I*100​​}\%\text{​} \end{array}$$

where *I* is the feeding amount (the weight of maize leaves before feeding minus the weight of maize leaves before feeding and after feeding); *B* is the average larval weight during the experiment (the average larval weight before feeding and after feeding); *T* is the experiment time (d); *G* is the added larval weight (the larval weight after feeding minus the larval weight before feeding); and *F* is the weight of total feces.

The larval lifespan, pupation rate/duration, and emergence of *M. separata* fed on leaves of *Bt* and non-*Bt* maize inoculated with and without *F. caledonium* were recorded every 12 h. Pairs of novel moths, including female: male ratio of 1: 1, were transferred to metal screen cages for oviposition and fed on 10% honey. The survivorship and fecundity of *M. separata* were observed every day until death.

### Data Analysis

Data were analyzed using IBM-SPSS v.20.0 software (IBM, Armonk, NY). Three-way ANOVAs were used to test the effects of sampling years (2017 vs. 2018), CO_2_ levels (elevated vs. ambient), AMF inoculation (*F. caledonium* vs. CK), transgenic treatment (*Bt* maize vs. non-*Bt* maize), and their interactions on the indices of Bt toxin and *Bt* gene expression in the leaves of *Bt* maize. Four-way ANOVAs were used to test the effects of sampling years (2017 vs. 2018), CO_2_ levels (elevated vs. ambient), AMF inoculation (*F. caledonium* vs. CK), transgenic treatment (*Bt* maize vs. non-*Bt* maize), and their interactions on the indices of AMF colonization and AMF-PLFA content, foliar contents of JA and SA, and growth, development, and reproduction of *M. separata*. Significant differences between or among treatments were analyzed by Tukey's test at *P* < 0.05.

## Results

### Arbuscular Mycorrhizal Fungi Colonization and AMF-PLFA Content of *Bt* and Non-*Bt* Maize Influenced by CO_2_ Levels and *F. caledonium* Inoculation

**Figure 1:**
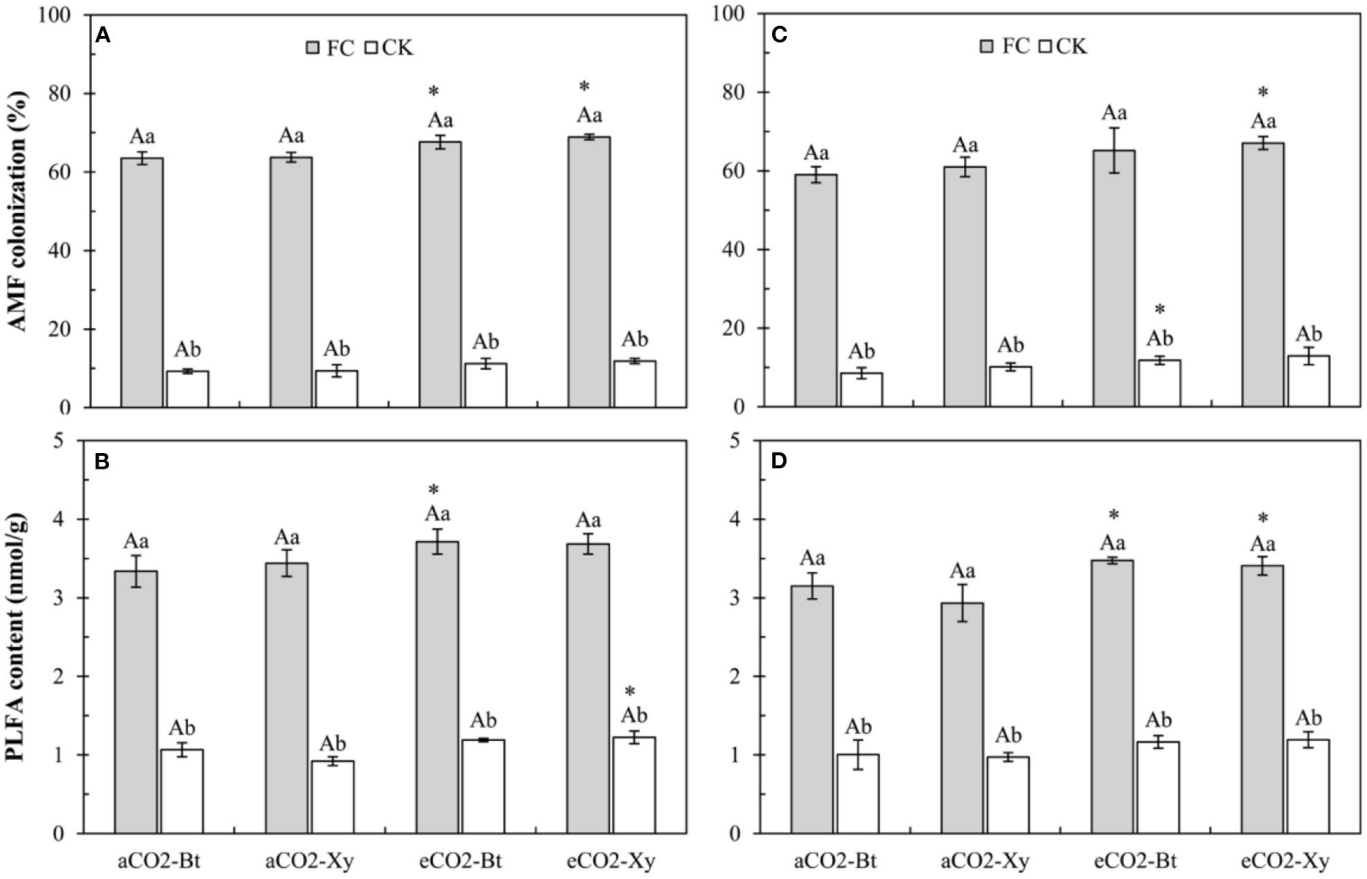
Effects of the inoculation with arbuscular mycorrhizal fungi (AMF), *F. caledonium*, on the AMF colonization and AMF-PLFA content of *Bt* maize (Bt) and its parental line of non-*Bt* maize (Xy) under ambient CO_2_ (*a*CO_2_) and elevated CO_2_ (*e*CO_2_) at the heading stage (BBCH-59) in 2017 **(A,B)** and 2018 **(C,D)**. (FC, *F. caledonium* inoculation; CK, the control without *F. caledonium* inoculation; *a*CO_2_-Bt, *Bt* maize under *a*CO_2_; *a*CO_2_-Xy, non-*Bt* maize under *a*CO_2_; *e*CO_2_-Bt, *Bt* maize under *e*CO_2_; *e*CO_2_-Xy, non-*Bt* maize under *e*CO_2_; different uppercase, lowercase letters, and * indicate significant difference between *Bt* maize and non-*Bt* maize, between *F. caledonium* inoculation and non-inoculation of *F. caledonium*, and between *a*CO_2_ and *e*CO_2_ under the same other conditions as revealed by Tukey's test) (*P* < 0.05; *n* = 15).

Four-way ANOVAs showed that AMF inoculation, CO_2_ level, and sampling years, and the interactions between AMF inoculation and CO_2_ level (*F* ≥ 4.47, *P* ≤ 0.042 < 0.05) significantly affected the AMF colonization on *Bt* and non-*Bt* maize (Supplementary Table 3). Compared with control, AMF inoculation significantly increased the AMF colonization of *Bt* and non-*Bt* maize in two sample years no matter under *a*CO_2_ or *e*CO_2_ (Figures 1A,C). Compared with *a*CO_2_, *e*CO_2_ significantly increased the AMF colonization of non-*Bt* maize inoculated with *F. caledonium* in 2017 and 2018, that of *Bt* maize inoculated with *F. caledonium* in 2017, and that of *Bt* maize not inoculated in 2018 (*P* < 0.05; Figures 1A,C).

Moreover, four-way ANOVAs also showed that AMF inoculation (*F* = 3385.14, *P* < 0.001), CO_2_ level (*F* = 52.68, *P* < 0.001), and sampling years (*F* = 17.27, *P* < 0.001) significantly affected the AMF-PLFA content of *Bt* and non-*Bt* maize (Supplementary Table 3). Compared with control, AMF inoculation significantly increased the AMF-PLFA content of *Bt* and non-*Bt* maize in two sample years no matter under *a*CO_2_ or *e*CO_2_ (Figures 1B,D). Compared with *a*CO_2_, *e*CO_2_ significantly increased the AMF-PLFA content of *Bt* maize inoculated with *F. caledonium* in 2017 and 2018, and also significantly increased the AMF-PLFA content of non-*Bt* maize inoculated with *F. caledonium* in 2018 and non-*Bt* maize not inoculated in 2017 (*P* < 0.05; Figures 1B,D).

### Foliar Bt Protein Content and *Bt* Gene Relative Expression Level in Leaves of *Bt* Maize Influenced by CO_2_ Levels and *F. caledonium* Inoculation

Three-way ANOVAs showed that AMF inoculation (*F* ≥ 275.07, *P* < 0.001), CO_2_ level (*F* ≥ 5.89, *P* ≤ 0.027), and their interaction (*F* ≥ 18.88, *P* < 0.001) significantly affected the foliar Bt protein content and *Bt* gene relative expression level in the leaves of *Bt* maize (Supplementary Table 4).

**Figure 2:**
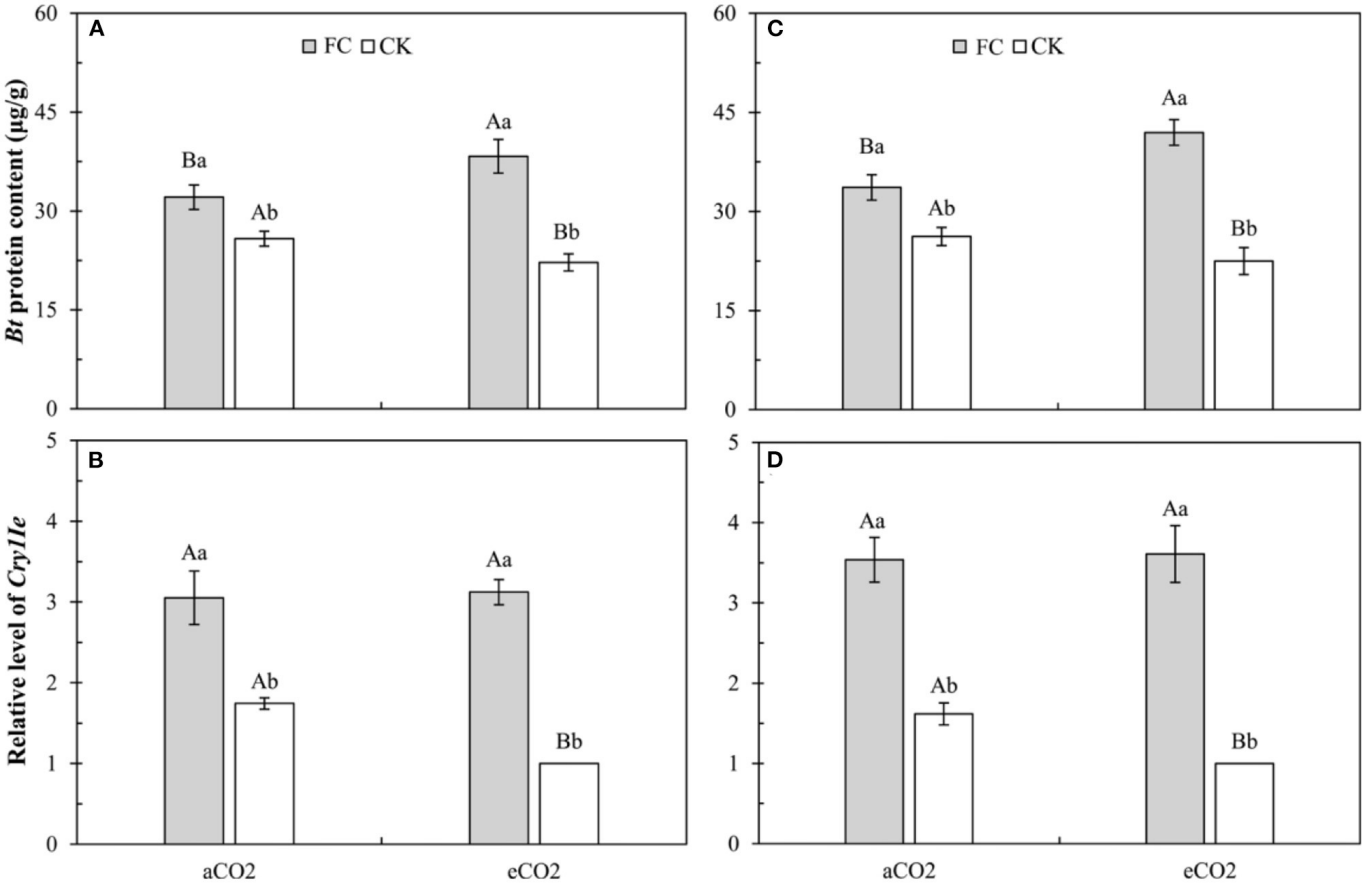
Effects of the inoculation with arbuscular mycorrhizal fungi (AMF), *F. caledonium*, on foliar Bt protein content and *Bt* gene relative expression level in leaves of *Bt* maize under ambient CO_2_ (*a*CO_2_) and elevated CO_2_ (*e*CO_2_) at the heading stage (BBCH-59) in 2017 **(A,B)** and 2018 **(C,D)**. (Note: Different uppercase and lowercase letters indicate a significant difference between *a*CO_2_ and *e*CO_2_, and between *F. caledonium* inoculation and non-inoculation of *F. caledonium* under the same other conditions as revealed by Tukey's test) (*P* < 0.05; *n* = 15).

For the foliar Bt protein content, compared with *a*CO_2_, *e*CO_2_ significantly decreased the foliar Bt protein content of *Bt* maize without *F. caledonium* inoculation, but significantly increased the foliar Bt protein content of *Bt* maize inoculated with *F. caledonium* (Figures 2A,C). Compared with control, AMF inoculation significantly increased the foliar Bt protein content of *Bt* maize under *a*CO_2_ and *e*CO_2_ (*P* < 0.05; Figures 2A,C).

For the *Bt* gene relative expression level, compared with *a*CO_2_, *e*CO_2_ significantly decreased the *Bt* gene relative expression level in the leaves of *Bt* maize without *F. caledonium* inoculation (Figures 2B,D). Compared with control, AMF inoculation significantly increased the *Bt* gene relative expression level of *Bt* maize under *a*CO_2_ and *e*CO_2_ (*P* < 0.05; Figures 2B,D).

### Foliar JA and SA Contents in Leaves of *Bt* and Non-*Bt* Maize Influenced by CO_2_ Levels and *F. caledonium* Inoculation

Four-way ANOVAs showed that AMF inoculation (*F* ≥ 216.16, *P* < 0.001) and CO_2_ level (*F* ≥ 99.02, *P* < 0.001) significantly affected the foliar JA and SA contents of *Bt* and non-*Bt* maize (Supplementary Table 4).

**Figure 3:**
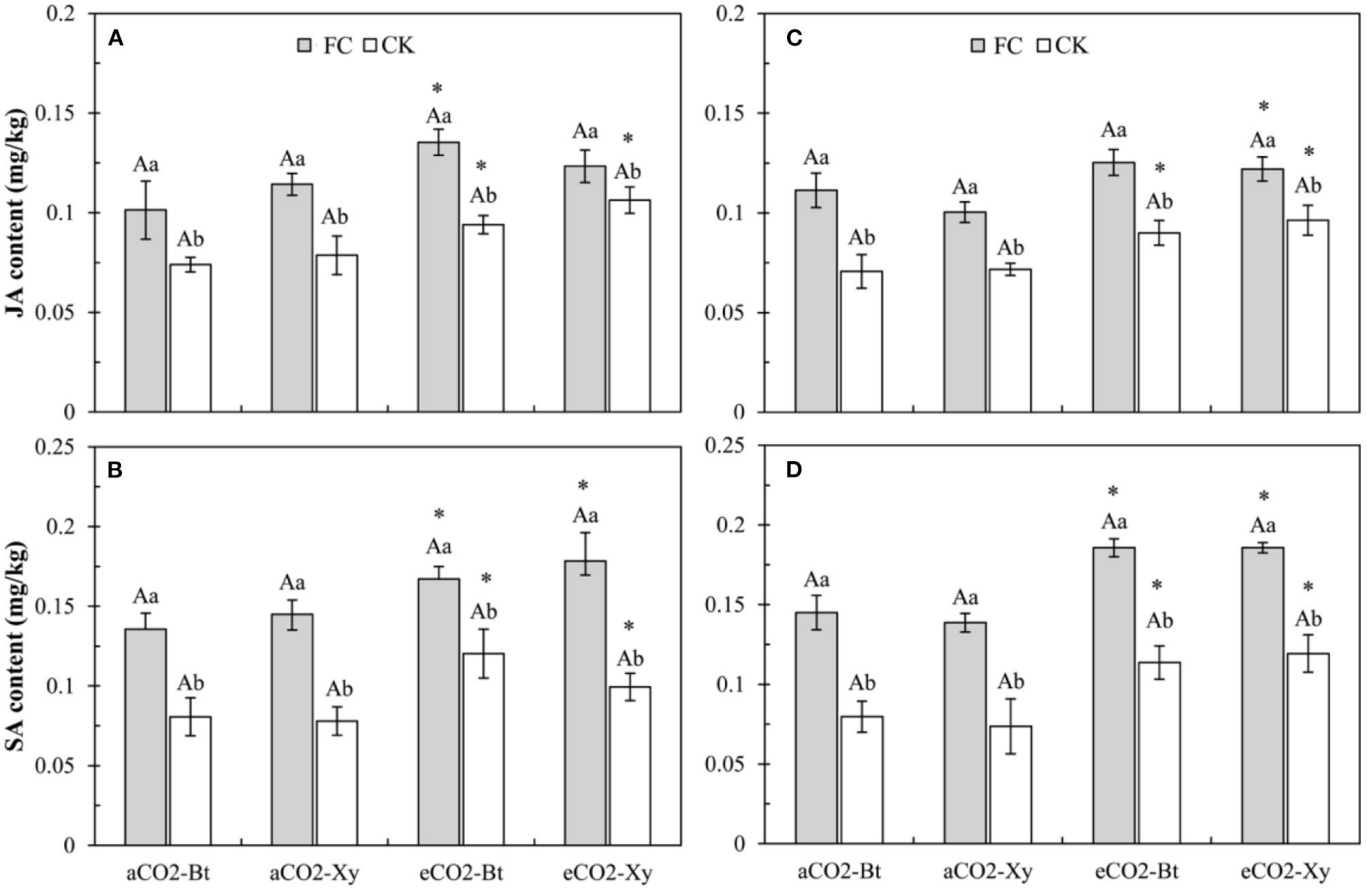
Effects of the inoculation with arbuscular mycorrhizal fungi (AMF), *F. caledonium*, on the foliar JA and SA contents of *Bt* maize (Bt) and its parental line of non-*Bt* maize (Xy) under ambient CO_2_ (*a*CO_2_) and elevated CO_2_ (*e*CO_2_) at the heading stage (BBCH-59) in 2017 **(A,B)** and 2018 **(C,D)**. (Note: FC, *F. caledonium* inoculation; CK, the control without *F. caledonium* inoculation; *a*CO_2_-Bt, *Bt* maize under *a*CO_2_; *a*CO_2_-Xy, non-*Bt* maize under *a*CO_2_; *e*CO_2_-Bt, *Bt* maize under *e*CO_2_; *e*CO_2_-Xy, non-*Bt* maize under *e*CO_2_; Different uppercase, lowercase letters and * indicate significant difference between *Bt* maize and non-*Bt* maize, between *F. caledonium* inoculation and non-inoculation of *F. caledonium*, and between *a*CO_2_ and *e*CO_2_ under the same other conditions as revealed by Tukey's test, the same in the following figures) (*P* < 0.05; *n* = 15).

For the foliar JA content, compared with control, AMF inoculation significantly increased the foliar JA content of *Bt* and non-*Bt* maize in two sample years no matter under *a*CO_2_ or *e*CO_2_ (Figures 3A,C). Compared with *a*CO_2_, *e*CO_2_ significantly increased the foliar JA content of *Bt* and non-*Bt* maize without *F. caledonium* inoculation in 2017 and 2018, and *Bt* maize inoculated with *F. caledonium* in 2017 and non-*Bt* maize inoculated with *F. caledonium* in 2018 (*P* < 0.05; Figures 3A,C).

For the foliar SA content, compared with control, AMF inoculation significantly increased the foliar SA content of *Bt* and non-*Bt* maize in two sample years no matter under *a*CO_2_ or *e*CO_2_ (Figures 3B,D). Compared with *a*CO_2_, *e*CO_2_ significantly increased the foliar SA content of *Bt* and non-*Bt* maize in two sample years regardless of *F. caledonium* inoculation or not (Figures 3B,D).

### Food Utilization of *M. separata* Larvae Fed on *Bt* and Non-*Bt* Maize Influenced by CO_2_ Levels and *F. caledonium* Inoculation

Four-way ANOVAs showed that AMF inoculation (*F* ≥ 4.20, *P* ≤ 0.048), CO_2_ level (*F* ≥ 4.24, *P* ≤ 0.047), transgenic treatment (*F* ≥ 36.98, *P* < 0.001), and their interactions (*F* ≥ 12.19, *P* < 0.0014) significantly affected all the food utilization indices of *M. separata* larvae, except for the interaction between CO_2_ level and AMF inoculation on RGR (*F* = 0.72, *P* = 0.40 > 0.05; Supplementary Table 5). Moreover, the interaction between CO_2_ level, AMF inoculation, and transgenic treatment also significantly affected the ECD (*F* = 32.63, *P* < 0.001) and ECI (*F* = 9.78, *P* = 0.004 < 0.01) of *M. separata* larvae (Supplementary Table 5).

**Figure 4:**
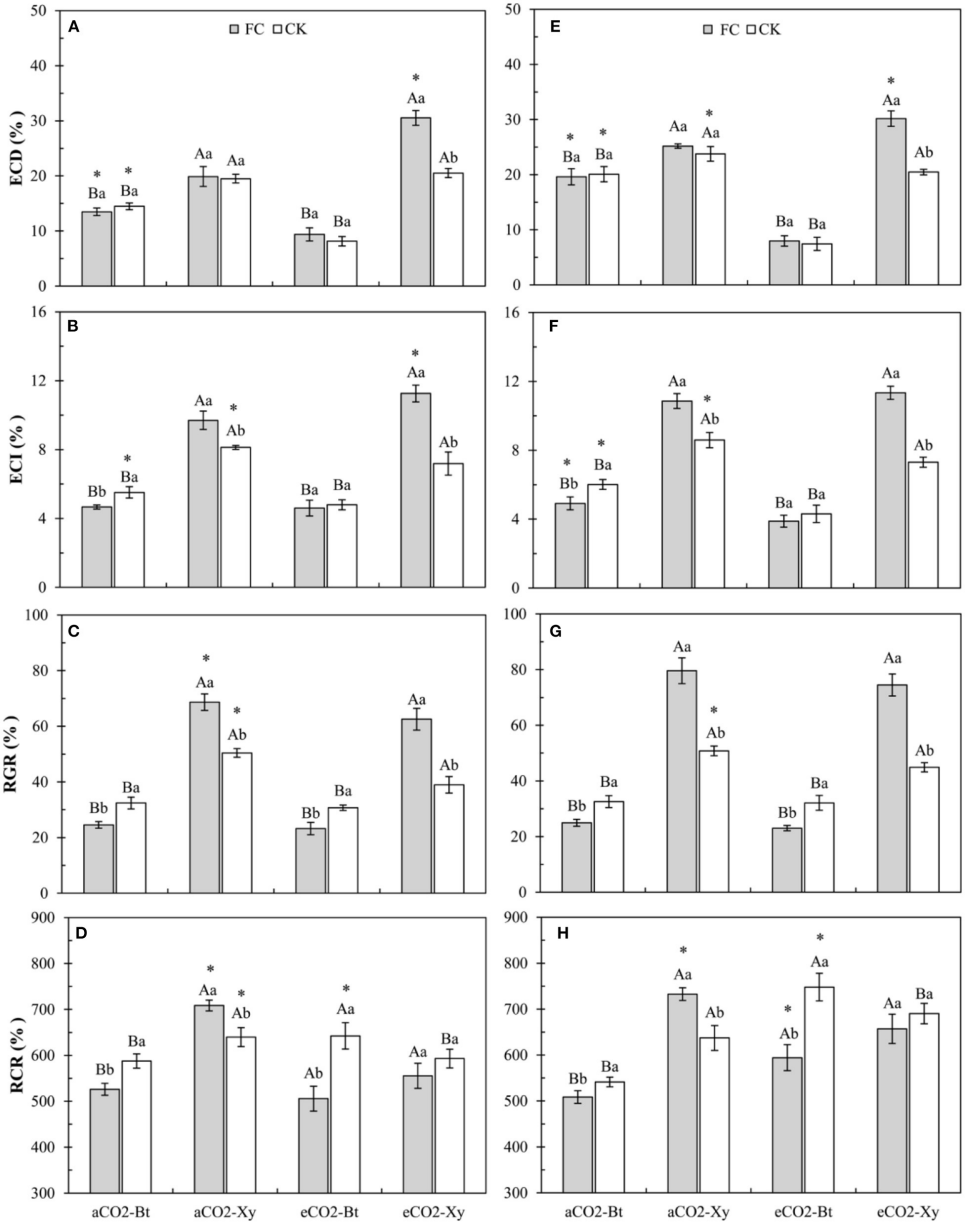
Effects of the inoculation with arbuscular mycorrhizal fungi (AMF), *F. caledonium*, on the food utilization of *M. separata* larvae fed on *Bt* maize (Bt) and its parental line of non-*Bt* maize (Xy) under ambient CO_2_ (*a*CO_2_) and elevated CO_2_ (*e*CO_2_) at the heading stage (BBCH-59) in 2017 **(A–D)** and 2018 **(E–H)**. (Note: the same as Figure 3) (*P* < 0.05; *n* = 5).

Compared with non-*Bt* maize, the ECD of *M. separata* larvae fed on *Bt* maize inoculated with and without *F. caledonium* was significantly decreased in two sample years no matter under *a*CO_2_ or *e*CO_2_. Compared with control, AMF inoculation significantly increased the ECD of *M. separata* larvae fed on non-*Bt* maize under *e*CO_2_ in 2017 and 2018. Compared with *a*CO_2_, *e*CO_2_ significantly decreased the ECD of *M. separata* larvae fed on *Bt* maize inoculated with and without *F. caledonium* in two sample years, while significantly increased the ECD of *M. separata* larvae fed on non-*Bt* maize inoculated with *F. caledonium* in 2017 and 2018, and significantly decreased the ECD of *M. separata* larvae fed on non-*Bt* maize without *F. caledonium* inoculation in 2018 (*P* < 0.05; Figures 4A,E).

Compared with non-*Bt* maize, the ECI of *M. separata* larvae fed on *Bt* maize inoculated with and without *F. caledonium* was significantly decreased in two sample years no matter under *a*CO_2_ or *e*CO_2_. Compared with control, AMF inoculation significantly increased the ECI of *M. separata* larvae fed on non-*Bt* maize under *a*CO_2_ and *e*CO_2_ in two sample years, while significantly decreased the ECI of *M. separata* larvae fed on *Bt* maize under *a*CO_2_ in 2017 and 2018. Compared with *a*CO_2_, *e*CO_2_ significantly decreased the ECI of *M. separata* larvae fed on *Bt* and non-*Bt* maize without *F. caledonium* inoculation in 2017 and 2018, while significantly increased the ECI of *M. separata* larvae fed on non-*Bt* maize with *F. caledonium* inoculation in 2017 and significantly decreased the ECI of *M. separata* larvae fed on *Bt* maize with *F. caledonium* inoculation in 2018 (*P* < 0.05; Figures 4B,F).

Compared with non-*Bt* maize, the RGR of *M. separata* larvae fed on *Bt* maize inoculated with and without *F. caledonium* was significantly decreased in two sample years no matter under *a*CO_2_ or *e*CO_2_. Compared with control, AMF inoculation significantly increased the RGR of *M. separata* larvae fed on non-*Bt* maize under aCO_2_ and *e*CO_2_ in 2017 and 2018, while significantly decreased the RGR of *M. separata* larvae fed on *Bt* maize under *a*CO_2_ and *e*CO_2_ in two sample years. Compared with *a*CO_2_, *e*CO_2_ significantly decreased the RGR of *M. separata* larvae fed on non-*Bt* maize without *F. caledonium* inoculation in 2017 and 2018, and that of *M. separata* larvae fed on non-*Bt* maize with *F. caledonium* inoculation in 2017 (*P* < 0.05; Figures 4C,G).

Compared with non-*Bt* maize, the RCR of *M. separata* larvae fed on *Bt* maize inoculated with and without *F. caledonium* was significantly decreased under *a*CO_2_ in 2017 and 2018, while the RCR of *M. separata* larvae fed on *Bt* maize without *F. caledonium* inoculation significantly increased under *e*CO_2_ in two sample years. Compared with control, AMF inoculation significantly decreased the RCR of *M. separata* larvae fed on *Bt* maize in two sample years no matter under *a*CO_2_ or *e*CO_2_, while significantly increased the RCR of *M. separata* larvae fed on non-*Bt* maize under *a*CO_2_ in 2017 and 2018. Compared with *a*CO_2_, *e*CO_2_ significantly decreased the RCR of *M. separata* larvae fed on non-*Bt* maize inoculated with *F. caledonium* in 2017 and 2018, while significantly increased the RCR of *M. separata* larvae fed on *Bt* maize without *F. caledonium* inoculation in two sample years (*P* < 0.05; Figures 4D,H).

### Growth, Development, and Reproduction of *M. separata* Fed on *Bt* and Non-*Bt* Maize Influenced by CO_2_ Levels and *F. caledonium* Inoculation

Four-way ANOVAs showed that sampling year (*F* ≥ 7.97, *P* ≤ 0.008), CO_2_ level (*F* ≥ 24.20, *P* < 0.001), transgenic treatment (*F* ≥ 164.88, *P* < 0.001), and the interaction between transgenic treatment and AMF inoculation (*F* ≥ 4.23, *P* ≤ 0.047) significantly affected all measured indices of *M*. *separata*, except the interaction between transgenic treatment and AMF inoculation on pupation rate of *M*. *separata* (*F* = 0.23, *P* = 0.63; Supplementary Table 6). Moreover, AMF inoculation significantly affected pupal weight and pupal duration of *M*. *separata* (*F* ≥ 15.07, *P* < 0.001), the interaction between CO_2_ level and transgenic treatment significantly affected larval lifespan of *M*. *separata* (*F* = 89.64, *P* < 0.001), and the interactions between CO_2_ level and AMF inoculation affected fecundity of *M*. *separata* (*F* = 12.65, *P* = 0.001) (Supplementary Table 6).

**Figure 5:**
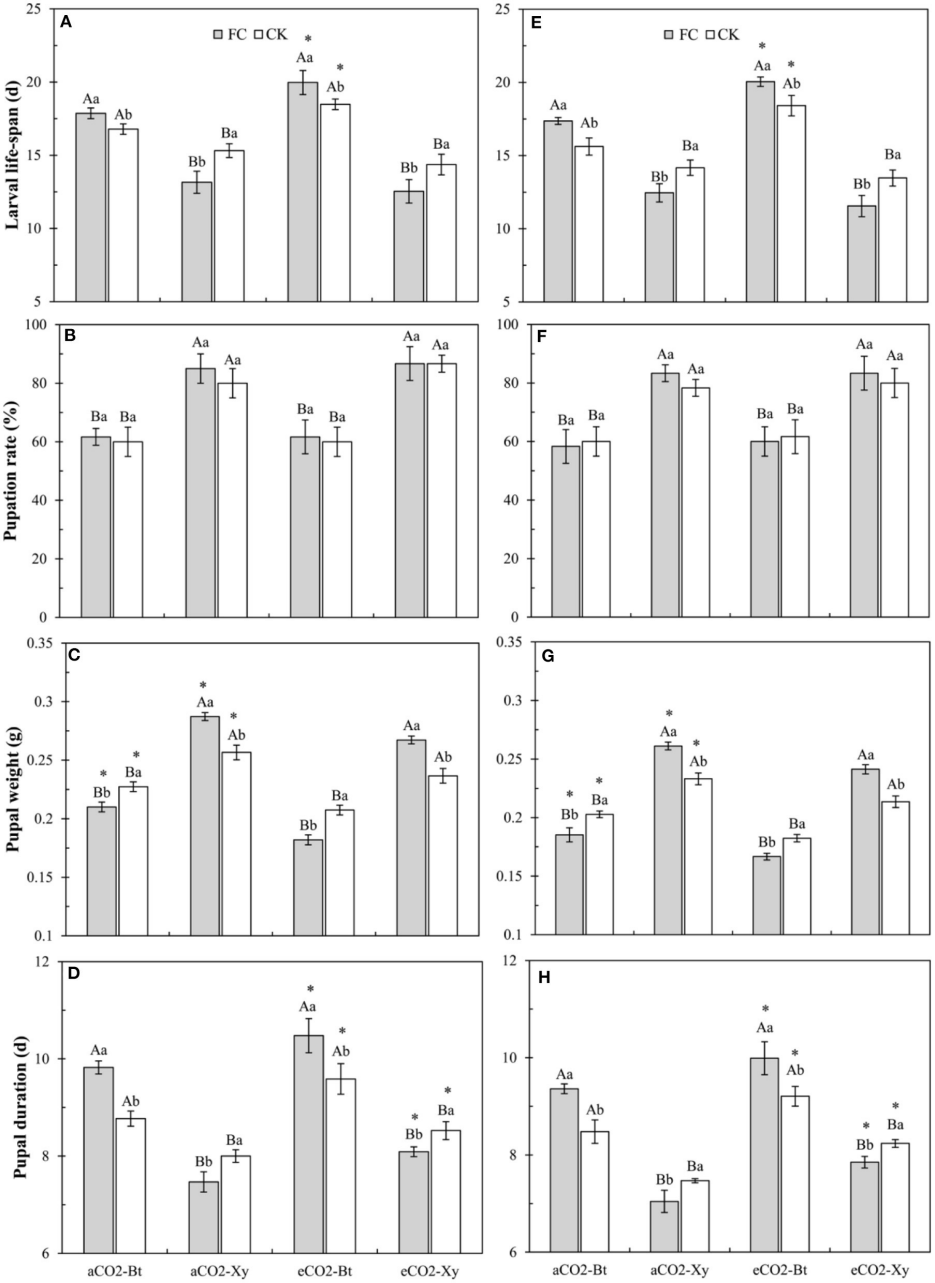
Effects of the inoculation with arbuscular mycorrhizal fungi (AMF), *F. caledonium*, on the growth and development of *M. separata* larvae fed on *Bt* maize (Bt) and its parental line of non-*Bt* maize (Xy) under ambient CO_2_ (*a*CO_2_) and elevated CO_2_ (*e*CO_2_) at the heading stage (BBCH-59) in 2017 **(A–D)** and 2018 **(E–H)**. (Note: the same as Figure 3) (*P* < 0.05; *n* = 5).

Compared with non-*Bt* maize, the larval lifespan of *M. separata* larvae fed on *Bt* maize inoculated with and without *F. caledonium* was significantly extended in two sample years no matter under *a*CO_2_ or *e*CO_2_. Compared with control, AMF inoculation significantly shortened the larval lifespan of *M. separata* fed on non-*Bt* maize under *a*CO_2_ and *e*CO_2_ in 2017 and 2018, while significantly prolonged the larval lifespan of *M. separata* fed on *Bt* maize under *a*CO_2_ and *e*CO_2_ in 2017 and 2018. Compared with *a*CO_2_, *e*CO_2_ significantly extended the larval lifespan of *M. separata* larvae fed on *Bt* maize inoculated with and without *F. caledonium* in two sample years (*P* < 0.05; Figures 5A,E).

Compared with non-*Bt* maize, the pupation rate of *M. separata* fed on *Bt* maize inoculated with and without *F. caledonium* was significantly decreased under *a*CO_2_ in two sample years no matter under *a*CO_2_ or *e*CO_2_ (*P* < 0.05; Figures 5B,F).

Compared with non-*Bt* maize, the pupal weight of *M. separata* fed on *Bt* maize inoculated with and without *F. caledonium* was significantly decreased in two sample years no matter under *a*CO_2_ or *e*CO_2_. Compared with control, AMF inoculation significantly increased the pupal weight of *M. separata* fed on non-*Bt* maize under *a*CO_2_ and *e*CO_2_ in 2017 and 2018, while significantly decreased the pupal weight of *M. separata* fed on *Bt* maize under *a*CO_2_ and *e*CO_2_ in two sample years. Compared with *a*CO_2_, *e*CO_2_ significantly decreased the pupal weight of *M. separata* fed on *Bt* and non-*Bt* maize in two sample years regardless of *F. caledonium* inoculation or not (*P* < 0.05; Figures 5C,G), respectively.

Compared with non-*Bt* maize, the pupal duration of *M. separata* fed on *Bt* maize inoculated with and without *F. caledonium* was significantly extended in two sample years no matter under *a*CO_2_ or *e*CO_2_. Compared with control, AMF inoculation significantly shortened the pupal duration of *M. separata* fed on non-*Bt* maize under *a*CO_2_ and *e*CO_2_ in 2017 and 2018, while significantly extended the pupal duration of *M. separata* fed on *Bt* maize under *a*CO_2_ and *e*CO_2_ in two sample years. Compared with *a*CO_2_, *e*CO_2_ significantly extended the pupal duration of *M. separata* fed on *Bt* and non-*Bt* maize in two sample years regardless of *F. caledonium* inoculation or not (*P* < 0.05; Figures 5D,H).

**Figure 6:**
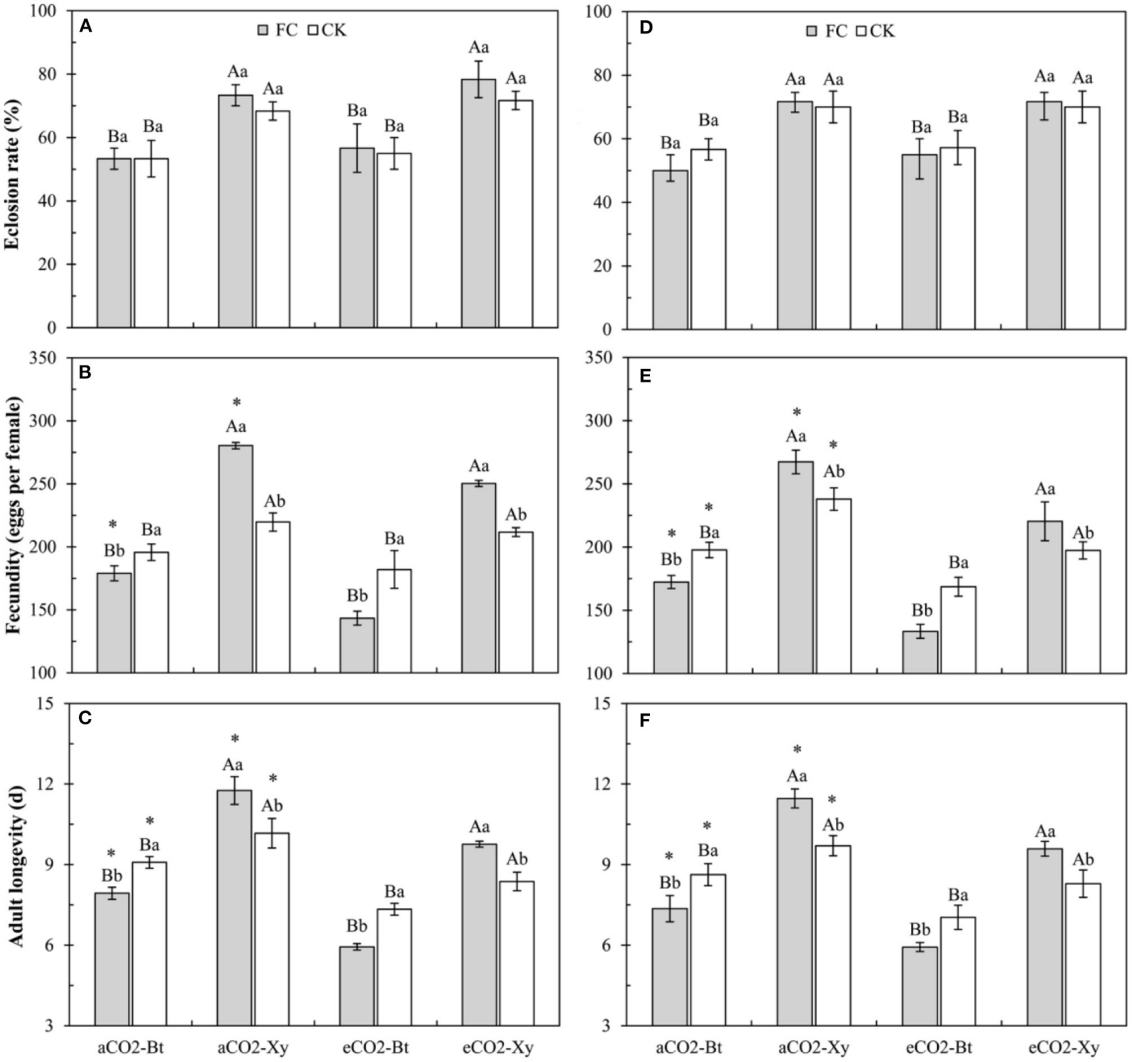
Effects of the inoculation with arbuscular mycorrhizal fungi (AMF), *F. caledonium*, on the growth and development of *M. separata* adults fed on *Bt* maize (Bt) and its parental line of non-*Bt* maize (Xy) under ambient CO_2_ (*a*CO_2_) and elevated CO_2_ (*e*CO_2_) at the heading stage (BBCH-59) in 2017 **(A–C)** and 2018 **(D–F)**. (Note: the same as Figure 3) (*P* < 0.05; *n* = 5).

Compared with non-*Bt* maize, the eclosion rate of *M. separata* fed on *Bt* maize inoculated with and without *F. caledonium* was significantly decreased in two sample years no matter under *a*CO_2_ or *e*CO_2_ (*P* < 0.05; Figures 6A,D).

Compared with non-*Bt* maize, the fecundity of *M. separata* fed on *Bt* maize inoculated with and without *F. caledonium* was significantly decreased in two sample years no matter under *a*CO_2_ or *e*CO_2_. Compared with control, AMF inoculation significantly increased the fecundity of *M. separata* fed on non-*Bt* maize under *a*CO_2_ and *e*CO_2_ in 2017 and 2018, while significantly decreased the fecundity of *M. separata* fed on *Bt* maize under *a*CO_2_ and *e*CO_2_ in two sample years. Compared with *a*CO_2_, *e*CO_2_ significantly decreased the fecundity of *M. separata* fed on *Bt* and non-*Bt* maize in two sample years regardless of *F. caledonium* inoculation or not (*P* < 0.05; Figures 6B,E).

Compared with non-*Bt* maize, the adult longevity of *M. separata* fed on *Bt* maize inoculated with and without *F. caledonium* was significantly shortened in two sample years no matter under *a*CO_2_ or *e*CO_2_. Compared with control, AMF inoculation significantly extended the adult longevity of *M. separata* fed on non-*Bt* maize under *a*CO_2_ and *e*CO_2_ in 2017 and 2018, while significantly shortened the adult longevity of *M. separata* fed on *Bt* maize under *a*CO_2_ and *e*CO_2_ in two sample years. Compared with *a*CO_2_, *e*CO_2_ significantly shortened the adult longevity of *M. separata* fed on *Bt* and non-*Bt* maize in two sample years regardless of *F. caledonium* inoculation or not (*P* < 0.05; Figures 6C,F).

### Yield of *Bt* and Non-*Bt* Maize Influenced by CO_2_ Levels and *F. caledonium* Inoculation

Four-way ANOVAs showed that AMF inoculation significantly affected all indices of maize yield (*F* ≥ 10.23, *P* ≤ 0.003), CO_2_ level significantly affected ear weight per plant and grain weight per ear (CO_2_ level: *F* ≥ 45.24, *P* < 0.001), transgenic treatment significantly affected ear weight per plant (*F* = 10.89, *P* = 0.002), and the interaction between CO_2_ level and AMF inoculation significantly affected the grain weight per ear (*F* = 4.48, *P* = 0.042 < 0.05) (Supplementary Table 3).

**Figure 7:**
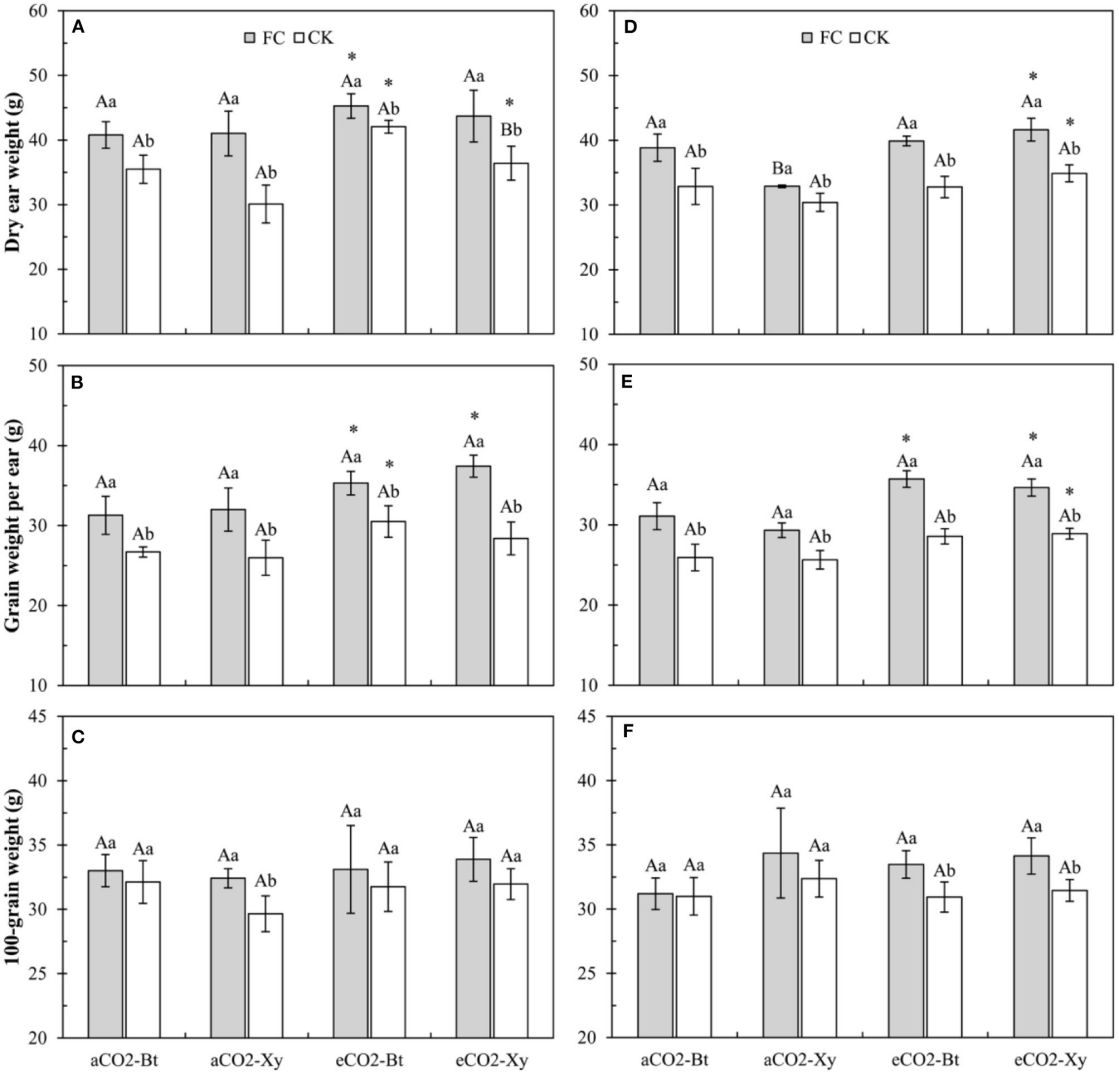
Effects of the inoculation with arbuscular mycorrhizal fungi (AMF), *F. caledonium*, on the yield of *Bt* maize (Bt) and its parental line of non-*Bt* maize (Xy) under ambient CO_2_ (*a*CO_2_) and elevated CO_2_ (*e*CO_2_) in 2017 **(A–C)** and 2018 **(D–F)**. (Note: the same as Figure 3) (*P* < 0.05; *n* = 15).

Compared with non-*Bt* maize, the dry ear weight per plant of *Bt* maize without *F. caledonium* inoculation was significantly enhanced under eCO_2_ in 2017, and that with *F. caledonium* inoculation was significantly increased under *a*CO_2_ in 2018. Compared with control, AMF inoculation significantly increased the dry ear weight per plant of *Bt* and non-*Bt* maize in two sample years no matter under *a*CO_2_ or *e*CO_2_. Compared with *a*CO_2_, *e*CO_2_ significantly increased the dry ear weight per plant of *Bt* and non-*Bt* maize in two sample years regardless of *F. caledonium* inoculation or not (*P* < 0.05; Figures 7A,D).

Compared with control, AMF inoculation significantly increased the grain weight per ear of *Bt* and non-*Bt* maize in two sample years no matter under *a*CO_2_ or *e*CO_2_. Compared with *a*CO_2_, *e*CO_2_ significantly increased the grain weight per ear of *Bt* and non-*Bt* maize inoculated with *F. caledonium* in two sample years, and significantly increased that of *Bt* maize without *F. caledonium* inoculation in 2017 and that of non-*Bt* maize without *F. caledonium* inoculation in 2018 (*P* < 0.05; Figures 7B,E). Compared with control, AMF inoculation significantly increased the 100-grain weight of non-*Bt* maize under *a*CO_2_ in 2017, and that of *Bt* and non-*Bt* maize under *e*CO_2_ in 2018 (*P* < 0.05; Figures 7C,F).

## Discussion

The values of AMF colonization and AMF-PLFA content indicate the colonization efficiency of AMF on maize. In this experiment, *F. caledonium* inoculation significantly increased the AMF colonization and AMF-PLFA content no matter what maize variety or CO_2_ level, and this ensured the validity of the following research work. Meanwhile, elevated CO_2_ can increase photosynthesis of plants, improve plant growth, and promote the transfer of carbon source substances from host plants to the root-symbiotic AMF, which is beneficial for AMF colonization and growth (Diaz et al., 1993; Cheng et al., 2012). The colonization was increased by ~10% in *Medicago truncatula* and by as much as 50% in *Brachypodium distachyon* (Jakobsen et al., 2016). Treseder (2004) also reported that the AMF colonization reflected an increased colonization speed under *e*CO_2_. Alberton et al. (2005) found that mycorrhizal fungi and mycorrhizal plants to elevated CO_2_ were significantly positive, and the response ratio for AM fungi was 1.21 (an increase of 21%), indicating a significantly different response, and AM colonization percentage also had a certain degree of improvement. In this study, the AMF colonization and the AMF-PLFA content of *Bt* and non-*Bt* maize under *e*CO_2_ were generally higher than those under *a*CO_2_; therefore, it is presumed that elevated CO_2_ did have a positive facilitation on the colonization of *F. caledonium* on maize (Diaz et al., 1993; Drigo et al., 2013; Becklin et al., 2016). Besides, there were no differences in the AMF colonization and AMF-PLFA content between *Bt* maize and its parental line of non-*Bt* maize; the results showed that the presence of the cry1Ie protein in maize did not affect the colonization of the AMF community, and it is consistent with the research report of Cheeke et al.; Cheeke et al. (2014; 2015; Zeng et al., 2018).

Overall, the plant biomass and grain yield increased with the increasing level (200–400 ppm) of atmospheric CO_2_ for most crops (Chen M. et al., 2011; Wang et al., 2018). In this study, the results showed that *e*CO_2_ significantly increased maize ear weight per plant and grain weight per ear, while it did not significantly affect the 100-grain weight. It is mainly because *e*CO_2_ can enhance photosynthesis and in turn, has a positive effect on crop biomass and production (Guo et al., 2016; Liu et al., 2019). Although the maize yield was improved, the comprehensive nutritional quality of maize grain (100-grain weight) could not be improved; it may be due to the decreased nitrogen content at high CO_2_ levels. Moreover, *F. caledonium* inoculation significantly increased all the yield indices (ear weight per plant, grain weight per ear, and 100-grain weight). The main function of AMF is to enhance the uptake of nutrient elements (e.g., N, P, K, Ca, Mg, Zn, and Fe) by host plants, improve the nutrient metabolism capacity and nutrient level of plant tissues, and then promote the growth and fruiting of plants (Sharifi et al., 2007; Terrer et al., 2016; Turrini et al., 2017). In addition, under the combined effects of *e*CO_2_ and *F. caledonium* inoculation, the ear weight per plant and grain weight per ear of *Bt* and non-*Bt* maize showed a further significant increase, which benefit from the improvement of AMF colonization under *e*CO_2_.

Insects are sensitive to environmental variations, and environmental stresses can cause changes in their growth, development, fecundity, food utilization, and the occurrence and distribution of populations as a result of metabolic rate fluctuation (Bloom et al., 2010). Usually, endogenously secondary defensive chemicals (e.g., JA and SA) and nutrient components (e.g., C, N, P, and K) are the two main factors that affect the population fitness of pests; the balance between secondary defensive chemicals and nutrient components determines the development trend of pests after feeding. If the pests feed on transgenic *Bt* plants, in addition to the above-mentioned two influencing factors, the Bt toxin protein will also have a significant adverse effect on the growth and food utilization of pests and occupy the dominant position among the three. Prutz and Dettner (2005) reported that the transgenic *Bt* maize decreased the growth rate and increased the mortality of *Chilo partellus*, which might be attributed to the termination of larval metamorphosis fed on *Bt* maize. Most studies showed adverse effects of *Cry* proteins on lifetable parameters of different herbivores (Lawo et al., 2010), which might be due to the interaction of feeding inhibitors and growth inhibitors. In this study, we found that *Bt* maize obviously decreased almost all measured indices of food utilization (ECD, ECI, and RGR), and it showed that the ability of food digestion and absorption of pests has caused serious damage by Bt toxin. Meanwhile, lifetable parameters (growth, development, and reproduction of *M. separata* larvae; pupation rate, pupal weight, eclosion rate, and fecundity of *M. separata* adult) markedly decreased feeding on *Bt* maize, and Bt toxin also prolonged the larval lifespan and shortened the adult longevity of *M. separata* regardless of the CO_2_ level and *F. caledonium* inoculation or not in 2017 and 2018. These results showed that *Bt* maize obviously retarded the growth and development of *M. separata* and were similar to those of previous studies.

Previous studies have examined that in most plants, elevated CO_2_ tends to promote plant photosynthesis and also leads to a decrease in foliar nitrogen content and an increase in C: N ratio (Johns et al., 2003; Li et al., 2018). Meanwhile, nitrogen is the main component of exogenous *Bt* protein in *Bt* crops. Plant nitrogen uptake, nitrogen-level status, and C: N ratio could affect the production of exogenous Bt toxins for *Bt* crops (Gao et al., 2009; Jiang et al., 2013). Numerous studies have shown that *e*CO_2_ can significantly reduce the exogenous Bt protein content of *Bt* cotton and *Bt* rice, while increasing their yield (Coviella et al., 2000, 2002; Chen et al., 2005b; Wu et al., 2011a,b), and also found the “dilution effect” on exogenous Bt protein or inhibition on *Bt*-transgene expression (Chen F. J. et al., 2011; Jiang et al., 2017; Liu et al., 2019). Moreover, elevated CO_2_ also affected the production of primary and secondary metabolites, and the defense mechanisms of crop plants (e.g., JA and SA) (Stiling and Cornelissen, 2007; Sun et al., 2016). In this study, *e*CO_2_ significantly decreased the ECI and RGR of *M. separata* larvae fed on non-*Bt* maize without *F. caledonium* inoculation, and was almost adverse to all the measured indices of growth and development of *M. separata*; it is mainly due to the increased secondary defense substances (i.e., JA and SA) in plants and the declined food nutrient level (e.g., fewer N) under elevated CO_2_ (Armstrong et al., 1995; Coviella et al., 2002; Liu et al., 2019). Many studies found that elevated CO_2_ had adverse effects on the developmental duration, pupation, and eclosion of cotton bollworm, *H. armigera* (Akbar et al., 2016), and reduced the oviposition number of borers and semilooper (Stange, 1997; Rao et al., 2013). On the other hand, elevated CO_2_ decreased the RGR of the *Spodoptera litura* and *H. armigera* (Hattenschwiler and Schafellner, 2004), and also significantly reduced the ECD and ECI of *H. armigera* (Yin et al., 2010). Meanwhile, *e*CO_2_ also significantly reduced the ECD, ECI, pupal weight, fecundity, and adult longevity, and significantly extended the development duration of *M. separata* fed on *Bt* maize with and without *F. caledonium* inoculation. This shows that despite the decrease in exogenous Bt toxin content, the increase in secondary defense substances and the decline in nutrient quality can also make a relatively adverse effect on *M. separata*.

The effect of *F. caledonium* inoculation on *M. separata* fed on *Bt* maize and non-*Bt* maize was just the opposite; that is, *F. caledonium* inoculation significantly reduced the ECI, RGR, and RCR of *M. separata* larvae fed on *Bt* maize under *a*CO_2_ and *e*CO_2_, while significantly increased the ECI and RGR of *M. separata* larvae fed non-*Bt* maize under *a*CO_2_ and *e*CO_2_, and RCR of *M. separata* larvae fed on non-*Bt* maize under *a*CO_2_, and ECD of *M. separata* larvae fed on non-*Bt* maize under *e*CO_2_. In addition, *F. caledonium* inoculation and transgenic treatment had a significant interaction on the growth, development, and reproduction of *M. separata*. Specifically, as fed on *Bt* maize, *F. caledonium* inoculation significantly extended the developmental duration of larvae and pupae of *M. separata* under *a*CO_2_ and *e*CO_2_, significantly reduced the pupal weight and fecundity, and significantly shortened the adult longevity of *M. separata*. On the contrary, as fed on non-*Bt* maize, *F. caledonium* inoculation significantly shortened the developmental duration of larvae and pupae of *M. separata* under *a*CO_2_ and *e*CO_2_, and significantly increased the pupal weight of *M. separata* under *a*CO_2_ and *e*CO_2_. Thus, it was observed that *F. caledonium* inoculation had diametrically opposite effects on the growth, development, and food utilization of *M. separata* fed on *Bt* maize and non-*Bt* maize; that is, *F. caledonium* inoculation reduced the food utilization efficiency and had adverse effects on the growth, development, and reproduction of *M. separata* fed on *Bt* maize, while it improved the food utilization efficiency and had positive effects on the growth, development, and reproduction of *M. separata* fed on non-*Bt* maize. This is mainly due to the promotion of the absorption and utilization of soil nutrients in maize plants by *F. caledonium* inoculation, thus improving the nutrient level of maize plant tissues (Sharifi et al., 2007; Rodriguez and Sanders, 2015). So it is concluded that *M. separata* larvae can get more plant nutrition when fed on non-*Bt* maize inoculated with *F. caledonium*. When fed on *Bt* maize inoculated with *F. caledonium, M. separata* larvae not only obtained more plant nutrients, but also ingested more doses of exogenous *Bt* toxin, which improved the target resistance ability of *Bt* crops based on the exogenous Bt toxin, and further reduced the food utilization efficiency, growth, development, and reproduction of *M. separata*, and at the same time, the promotion of AMF colonization by *e*CO_2_ further enhanced the target resistance level of *Bt* maize to *M. separata*.

**Figure 8:**
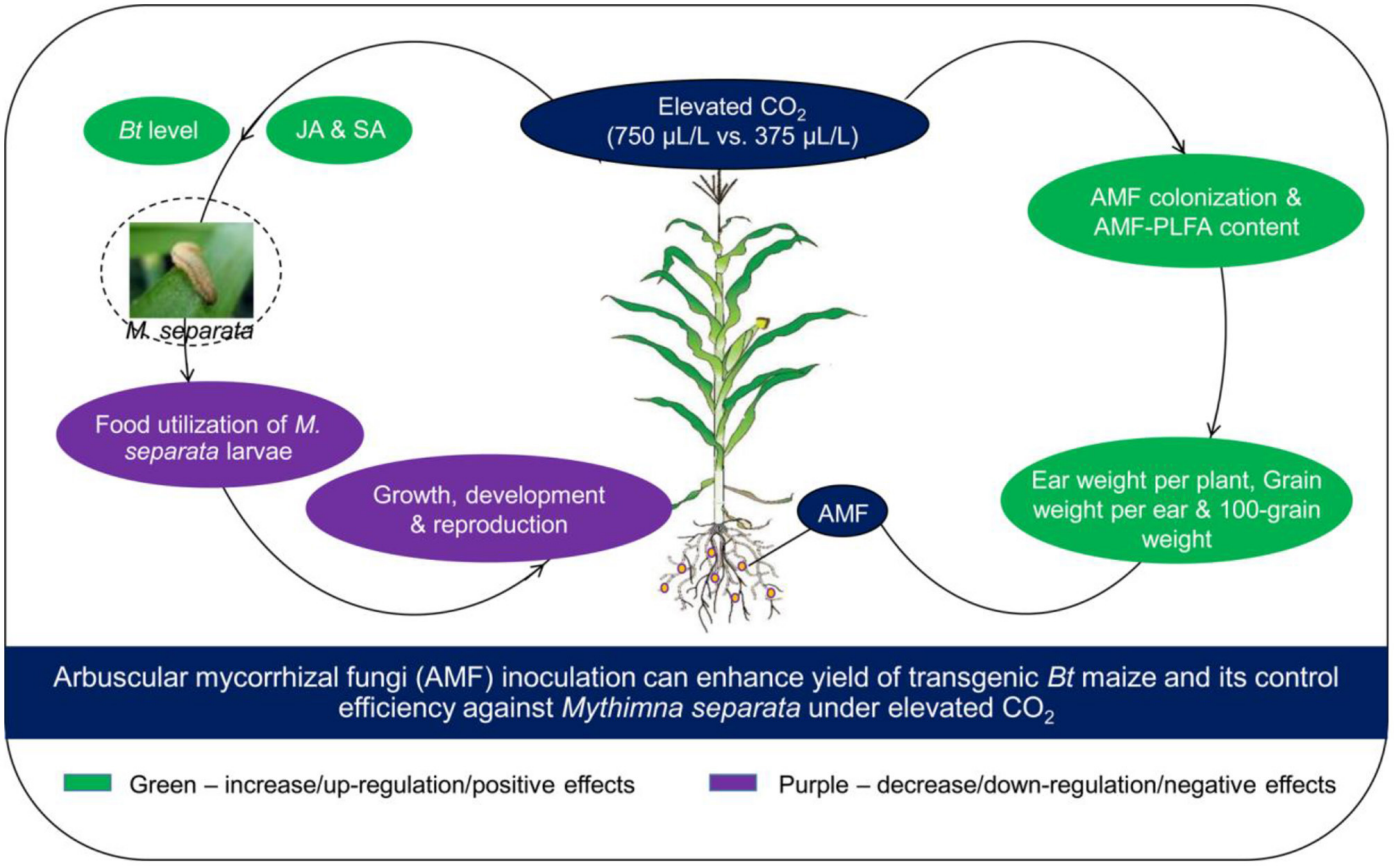
A schematic model that AMF inoculation can enhance the yield of transgenic *Bt* maize and its control efficiency against *M. separata* under elevated CO_2_.

Overall, our research showed that *e*CO_2_ was beneficial for AMF colonization on roots, and maize yield, but it had negative effects on the growth, development, reproduction, and food utilization of *M. separata*. *F. caledonium* inoculation was positive for maize yield and nutrient quality, and in favor of the growth, development, reproduction, and food utilization of the *M. separata* fed on non-*Bt* maize. However, *F. caledonium* inoculation was unfavorable for the population fitness of *M. separata* fed on *Bt* maize. Namely, regardless of the CO_2_ level, inoculation of *F. caledonium* had a detrimental effect on the production of non-*Bt* maize as the result of a high potential risk of *M. separata* production hazard, while its effects on *Bt* maize were just the opposite; that is, *F. caledonium* inoculation had positive effects on the production of *Bt* maize especially under *e*CO_2_ due to the lower potential risk of population occurrence of *M. separata*. Ultimately, the results proved that all of our hypotheses were confirmed: It showed that the AMF inoculation of *F. caledonium* under *e*CO_2_ was more effective in improving the control efficiency of *Bt* maize on the target insect pest, *M. separata*, promoted the expression of endogenous (JA, SA) and exogenous (Bt toxin) secondary defense substances in *Bt* maize leaves, and also increased the biomass and yield of maize (Figure 8). We have reason to expect this friendly and effective biological way serving for mitigating the ecological risk of *Bt* maize and improving its ecological sustainable utilization capacity under global climate change.

## Data Availability Statement

The original contributions presented in the study are included in the article/Supplementary Materials, further inquiries can be directed to the corresponding author/s.

## Author Contributions

LW and FC conceived research. LW, XW, and TH conducted the experiments. LW, FG, CL, and LL analyzed data and conducted statistical analysis. LW and FC wrote the manuscript. FC secured funding. All authors read and approved the manuscript.

## Conflict of Interest

The authors declare that the research was conducted in the absence of any commercial or financial relationships that could be construed as a potential conflict of interest.
