# Supplementary material for: AMF Inoculation Can Enhance Yield of Transgenic Bt Maize and Its Control Efficiency Against Mythimna separata Especially Under Elevated CO2
Source: Front Plant Sci. 2021 Jun 8;12:655060. doi: 10.3389/fpls.2021.655060 (PMC8217876; doi:10.3389/fpls.2021.655060)
Supplement: Supplementary file 1 [file Table_1.DOCX]

| **Supplementary table 1** Actual mean +/- SE CO_2_ level in the open-top chambers (OTCs) from seedling emergence to harvest of *Bt* maize and its parental line of non-*Bt* maize in 2017 and 2018 | | | |
| --- | --- | --- | --- |
| **Treatments** | **factors level** | **2017** | **2018** |
| CO_2_（μL/L） | Elevated-CO_2_ | 750.6 ± 4.5 a | 749.1 ± 3.7 a |
|  | Ambient-CO_2_ | 375.4 ± 3.8 b | 373.8 ± 5.2 b |
| **Note:** The values represent the means +/- SE. Different letters indicate significant differences between treatments within the same year according to Tukey test (*P* < 0.05), no differences were observed between years within the same treatment. | | | |
