# Supplementary material for: AMF Inoculation Can Enhance Yield of Transgenic Bt Maize and Its Control Efficiency Against Mythimna separata Especially Under Elevated CO2
Source: Front Plant Sci. 2021 Jun 8;12:655060. doi: 10.3389/fpls.2021.655060 (PMC8217876; doi:10.3389/fpls.2021.655060)
Supplement: Supplementary file 2 [file Table_2.DOCX]

| **Supplementary table 2** Primers of *Cry1Ie* genes used in the quantitative real-time PCR (qRT-PCR) | | | |
| --- | --- | --- | --- |
| **Primer** | **Sequence (5′→ 3′)** | **GeneBank** | **Description** |
| *Cry1Ie*-F | ACCATTAGTAAAAGCGTTCAATCTGT | AF211190.1 | *Bt* protein gene |
| *Cry1Ie*-R | ATCCCCAAATGTACCAGTATTCGTT |  |  |
| β-actin-F | GATTCCTGGGATTGCCGAT | J01238.1 | Reference gene |
| β-actin-R | TCTGCTGCTGAAAAGTGCTGAG |  |  |
