# Supplementary material for: AMF Inoculation Can Enhance Yield of Transgenic Bt Maize and Its Control Efficiency Against Mythimna separata Especially Under Elevated CO2
Source: Front Plant Sci. 2021 Jun 8;12:655060. doi: 10.3389/fpls.2021.655060 (PMC8217876; doi:10.3389/fpls.2021.655060)
Supplement: Supplementary file 3 [file Table_3.DOCX]

| **Supplementary table 3** Four-way ANOVAs for the effects of CO_2_ level, AMF inoculation, transgenic *Bt* treatment, sampling years and their interactions on the AMF colonization, AMF-PLFA content and the maize yields (*F*/*P* values) | | | | | |
| --- | --- | --- | --- | --- | --- |
| **Impact factors** | **AMF Colonization**  **(%)** | **AMF-PLFA content (nmol/g)** | **Ear weight per plant**  **(dry; g)** | **Grain weight per ear**  **(dry; g)** | **100-grain weight**  **(dry; g)** |
| Y^a^ | 4.47/0.042^*^ | 17.27/<0.001^***^ | 34.72/<0.001^***^ | 5.18/0.030^*^ | 0.01/0.91 |
| CO_2_^b^ | 45.98/<0.001^***^ | 52.68/<0.001^***^ | 45.24/<0.001^***^ | 72.10/<0.001^***^ | 0.61/0.44 |
| Cv.^c^ | 3.61/0.067 | 1.11/0.30 | 11.64/0.002^**^ | 0.27/0.61 | 1.45/0.24 |
| AMF^d^ | 8334.57/<0.001^***^ | 3585.14/<0.001^***^ | 92.47/<0.001^***^ | 142.21/<0.001^***^ | 10.23/0.003^**^ |
| Y × CO_2_ | 0.97/0.33 | 0.18/0.68 | 1.12/0.30 | 0.06/0.81 | 0.83/0.37 |
| Y × Cv. | 0.82/0.37 | 0.66/0.42 | 2.30/0.14 | 0.26/0.61 | 5.24/0.029^*^ |
| Y × AMF | 7.74/0.009^**^ | 13.85/0.001 | 0.54/0.47 | 0.85/0.36 | 0.03/0.86 |
| CO_2_ × Cv. | 0.04/0.84 | 0.70/0.41 | 4.77/0.036^*^ | 0.02/0.89 | 0.28/0.6 |
| CO_2_ × AMF | 5.48/0.026^*^ | 3.86/0.058 | 0.08/0.78 | 4.48/0.042^*^ | 0.13/0.73 |
| Cv. × AMF | 0.17/0.68 | 0.10/0.76 | 1.41/0.24 | 0.90/0.35 | 2.09/0.16 |
| Y× CO_2_ × Cv. | 0.20/0.66 | 0.26/0.61 | 7.50/0.01^*^ | 0.02/0.90 | 2.33/0.14 |
| Y × CO_2_ × AMF | 0.07/0.79 | 0.53/0.47 | 5.15/0.03^*^ | 0.19/0.67 | 0.24/0.63 |
| Y × Cv. × AMF | 0.01/0.92 | 2.27/0.14 | 7.55/0.01^*^ | 4.12/0.051 | 0.03/0.86 |
| CO_2_ × Cv. × AMF | 0.04/0.84 | 0.53/0.47 | 0.035/0.85 | 0.29/0.59 | 0.18/0.68 |
| Y× Cv. × CO_2_ × AMF | 0.001/0.98 | 1.78/0.19 | 0.87/0.36 | 0.87/0.36 | 0.08/0.79 |
| **Note:** ^*^*P*<0.05, ^**^*P*<0.01, ^***^*P*<0.001; ^a^: Years (2017 vs. 2018); ^b^: CO_2_ level (Elevated vs. Ambient); ^c^: Transgenic treatment (*Bt* maize vs. non-*Bt* maize); ^d^: AMF inoculation (*G. caledonium* vs. CK). | | | | | |
