# Supplementary material for: AMF Inoculation Can Enhance Yield of Transgenic Bt Maize and Its Control Efficiency Against Mythimna separata Especially Under Elevated CO2
Source: Front Plant Sci. 2021 Jun 8;12:655060. doi: 10.3389/fpls.2021.655060 (PMC8217876; doi:10.3389/fpls.2021.655060)
Supplement: Supplementary file 4 [file Table_4.DOCX]

| **Supplementary table 4** Three-way ANOVAs for the effects of CO_2_ level, AMF inoculation, sampling years and their interactions on the foliar *Bt* toxin content and *Bt* gene relative expression level in leaves of *Bt* maize, and four-way ANOVAs for the effects of CO_2_ level, AMF inoculation, transgenic *Bt* treatment, sampling years and their interactions on the foliar JA and SA contents in leaves of *Bt* maize and non-*Bt* maize (*F*/*P* values) | | | | |
| --- | --- | --- | --- | --- |
| **Impact factors** | ***Bt* protein content (μg/g)** | ***Bt* gene relative expression level** | **JA content (mg/kg)** | **SA content (mg/kg)** |
| Y^a^ | 3.91/0.066 | 5.94/0.027^*^  12.36/0.003** | 5.37/<0.027^*^ | 2.14/0.15 |
| CO_2_^b^ | 5.89/0.027^*^ | 12.36/0.003^**^ | 99.02/<0.001^***^ | 133.81/<0.001^***^ |
| Cv.^c^ |  |  | 0.41/<0.53 | 0.15/0.70 |
| AMF^d^ | 275.07/<0.001^***^ | 529.68/<0.001^***^ | 216.16/<0.001^***^ | 415.59/<0.001^***^ |
| Y × CO_2_ | 0.46/0.51  2.27/0.15 | 0.14/0.71 | 0.42/<0.52 | 2.70/0.11 |
| Y × Cv. |  |  | 2.13/0.60 | 0.02/0.88 |
| Y × AMF | 2.27/0.15 | 10.05/0.006^**^ | 0.28/0.15 | 0.69/0.42 |
| CO_2_ × Cv. |  |  | 0.06/0.80 | 0.005/0.95 |
| CO_2_ × AMF | 53.89/<0.001^***^ | 18.88/0.001^**^ | 0.58/0.45 | 0.21/0.65 |
| Cv. × AMF |  |  | 4.84/0.035^*^ | 2.30/0.14 |
| Y× CO_2_ × Cv. |  |  | 3.14/0.086 | 1.84/0.18 |
| Y × CO_2_ × AMF | 0.55/0.47 | 0.13/0.73 | 0.05/<0.83 | 0.03/0.86 |
| Y × Cv. × AMF |  |  | 0.11/0.74 | 3.94/0.056 |
| CO_2_ × Cv. × AMF |  |  | 3.15/0.085 | 0.35/0.56 |
| Y× Cv. × CO_2_ × AMF |  |  | 4.18/<0.049^*^ | 1.03/0.32 |
| **Note:** ^*^*P*<0.05, ^**^*P*<0.01, ^***^*P*<0.001; ^a^: Years (2017 vs. 2018); ^b^: CO_2_ level (Elevated vs. Ambient); ^c^: Transgenic treatment (*Bt* maize vs. non-*Bt* maize); ^d^: AMF inoculation (*G. caledonium* vs. CK). | | | | |
