# Supplementary material for: AMF Inoculation Can Enhance Yield of Transgenic Bt Maize and Its Control Efficiency Against Mythimna separata Especially Under Elevated CO2
Source: Front Plant Sci. 2021 Jun 8;12:655060. doi: 10.3389/fpls.2021.655060 (PMC8217876; doi:10.3389/fpls.2021.655060)
Supplement: Supplementary file 5 [file Table_5.DOCX]

| **Supplementary table 5** Four-way ANOVAs for the effects of CO_2_ level, AMF inoculation, transgenic *Bt* treatment, sampling years and their interactions on the food utilization indices of armyworm (*Mythimna separata*) from the 3rd to the 6th instar larvae (*F*/*P* values) | | | | |
| --- | --- | --- | --- | --- |
| **Impact factors** | **ECD (%)** | **ECI (%)** | **RGR (mg g^-1^day^-1^)** | **RCR (mg g^-1^day^-1^)** |
| Y^a^ | 53.50/<0.001^***^ | 2.16/0.15 | 27.60/<0.001^***^ | 45.28/<0.001^***^ |
| CO_2_^b^ | 69.23/<0.001^***^ | 15.75/<0.001^***^ | 33.34/<0.001^***^ | 4.24/0.047^*^ |
| Cv.^c^ | 1209.50/<0.001^***^ | 1459.63<0.001^***^ | 1754.72/<0.001^***^ | 115.56/<0.001^***^ |
| AMF^d^ | 71.94/<0.001^***^ | 101.08/<0.001^***^ | 134.04/<0.001^***^ | 31.21/<0.001^***^ |
| Y × CO_2_ | 85.66/<0.001^***^ | 13.04/0.001^**^ | 1.52/0.23 | 69.80/<0.001^***^ |
| Y × Cv. | 0.03/0.86 | 5.96/0.020^*^ | 21.80/<0.001^***^ | 3.04/0.091 |
| Y × AMF | 0.04/0.84 | 0.03/0.86 | 6.62/0.015^*^ | 0.68/0.42 |
| CO_2_ × Cv. | 349.76/<0.001^***^ | 12.90/0.001^**^ | 15.51/<0.001^***^ | 110.86/<0.001^***^ |
| CO_2_ × AMF | 67.33/<0.001^***^ | 35.81/<0.001^***^ | 0.72/0.40 | 68.23/<0.001^***^ |
| Cv. × AMF | 67.93/<0.001^***^ | 240.97/<0.001^***^ | 504.12/<0.001^***^ | 94.28/<0.001^***^ |
| Y× CO_2_ × Cv. | 2.27/0.14 | 0.31/0.58 | 1.02/0.32 | 2.42/0.13 |
| Y × CO_2_ × AMF | 1.02/0.32 | 0.61/0.44 | 1.18/0.28 | 1.68/0.20 |
| Y × Cv. × AMF | 0.12/0.73 | 1.46/0.24 | 9.19/0.005^**^ | 0.13/0.72 |
| CO_2_ × Cv. × AMF | 32.63/<0.001^***^ | 9.78/0.004^**^ | 1.52/0.23 | 0.56/0.46 |
| Y× Cv. × CO_2_ × AMF | 0.001/0.97 | 0.66/0.42 | 0.21/0.65 | 0.20/0.65 |
| **Note:** ^*^*P*<0.05, ^**^*P*<0.01, ^***^*P*<0.001; ^a^: Years (2017 vs. 2018); ^b^: CO_2_ level (Elevated vs. Ambient); ^c^: Transgenic treatment (*Bt* maize vs. non-*Bt* maize); ^d^: AMF inoculation (*G. caledonium* vs. CK); RCR (relative consumption rate), RGR (relative growth rate), ECD (efficiency of conversion of digested food), ECI (efficiency of conversion of ingested food). | | | | |
