# Supplementary material for: AMF Inoculation Can Enhance Yield of Transgenic Bt Maize and Its Control Efficiency Against Mythimna separata Especially Under Elevated CO2
Source: Front Plant Sci. 2021 Jun 8;12:655060. doi: 10.3389/fpls.2021.655060 (PMC8217876; doi:10.3389/fpls.2021.655060)
Supplement: Supplementary file 6 [file Table_6.DOCX]

| **Supplementary table 6** Four-way ANOVAs for the effects of CO_2_ level, AMF inoculation, transgenic *Bt* treatment, sampling years and their interactions on the growth, development and reproduction of armyworm (*Mythimna separata*) (*F/P* values) | | | | | | | |
| --- | --- | --- | --- | --- | --- | --- | --- |
| **Impact factors** | **Larval life-span**  **(day)** | **Pupation rate**  **(%)** | **Pupal weight**  **(%)** | **Pupal duration**  **(day)** | **Eclosion rate**  **(%)** | **Adult longevity**  **(day)** | **Fecundity**  **(eggs per female)** |
| Y^a^ | 19.06/<0.001^***^ | 3.10/0.088 | 343.48/<0.001^***^ | 43.55/<0.001^***^ | 0.53/0.47 | 7.97/0.008^**^ | 13.62/0.001^**^ |
| CO_2_^b^ | 24.20/<0.001^***^ | 2.08/0.16 | 268.71/<0.001^***^ | 140.01/<0.001^***^ | 3.10/0.088 | 273.57/<0.001^***^ | 180.28/<0.001^***^ |
| Cv.^c^ | 838.53/<0.001^***^ | 272.03/<0.001^***^ | 1821.18/<0.001^***^ | 773.34/<0.001^***^ | 164.88/<0.001^***^ | 563.31/<0.001^***^ | 762.43/<0.001^***^ |
| AMF^d^ | 1.24/0.27 | 2.08/0.16 | 16.31/<0.001^***^ | 15.07/<0.001^***^ | 0.52/0.48 | 1.80/0.19 | 0.43/0.52 |
| Y × CO_2_ | 1.19/0.28 | 0.23/0.63 | 0.91/0.35 | 0.48/0.49 | 0.53/0.47 | 2.14/0.15 | 14.35/0.001^**^ |
| Y × Cv. | 3.23/0.08 | 0.03/0.87 | 0.79/0.38 | 0.07/0.79 | 0.69/0.41 | 0.16/0.69 | 0.006/0.94 |
| Y × AMF | 0.53/0.47 | 0.23/0.63 | 0.16/0.70 | 0.06/0.81 | 3.10/0.088 | 0.09/0.76 | 6.30/0.017^*^ |
| CO_2_ × Cv. | 89.64/<0.001^***^ | 0.03/0.87 | 0.55/0.46 | 0.04/0.84 | 0.04/0.84 | 0.14/0.71 | 3.80/0.06 |
| CO_2_ × AMF | 0.28/0.60 | .023/0.63 | 0.41/0.52 | 0.08/0.78 | 0.52/0.48 | 0.82/0.37 | 12.65/0.001^**^ |
| Cv. × AMF | 107.07/<0.001^***^ | 0.23/0.63 | 361.24/<0.001^***^ | 132.16/<0.001^***^ | 4.23/0.047^*^ | 171.37/<0.001^***^ | 207.73/<0.001^***^ |
| Y× CO_2_ × Cv. | 2.31/0.14 | 0.03/0.87 | 0.71/0.41 | 1.28/0.27 | 0.69/0.41 | 0.06/0.80 | 1.47/0.23 |
| Y × CO_2_ × AMF | 1.04/0.31 | 0.03/0.87 | 0.88/0.36 | 0.002/0.97 | 0.01/0.92 | 0.03/0.86 | 1.66/0.21 |
| Y × Cv. × AMF | 0.29/0.60 | 3.10/0.088 | 2.14/0.15 | 0.80/0.38 | 0.04/0.83 | 0.01/0.91 | 2.31/0.14 |
| CO_2_ × Cv.× AMF | 0.003/0.957 | 2.08/0.0.16 | 0.34/0.56 | 0.79/0.38 | 0.18/0.67 | 0.48/0.50 | 3.07/0.089 |
| Y× Cv. × CO_2_ × AMF | 0.03/0.85 | 1.26/0.28 | 0.99/0.33 | 0.09/0.77 | 0.17/0.68 | 0.67/0.42 | 0.04/0.84 |
| **Note:** ^*^*P*<0.05, ^**^*P*<0.01, ^***^*P*<0.001; ^a^: Years (2017 vs. 2018); ^b^: CO_2_ level (Elevated vs. Ambient); ^c^: Transgenic treatment (*Bt* maize vs. non-*Bt* maize); ^d^: AMF inoculation (*G. caledonium* vs. CK). | | | | | | | |
